# Species identification of spider mites (Tetranychidae: Tetranychinae): a review of methods

**DOI:** 10.18699/VJGB-23-30

**Published:** 2023-06

**Authors:** A.V. Razuvaeva, E.G. Ulyanova, E.S. Skolotneva, I.V. Andreeva

**Affiliations:** Institute of Cytology and Genetics of the Siberian Branch of the Russian Academy of Sciences, Novosibirsk, Russia; Siberian Federal Scientific Centre of Agro-BioTechnologies of the Russian Academy of Sciences, Krasnoobsk, Novosibirsk Region, Russia; Institute of Cytology and Genetics of the Siberian Branch of the Russian Academy of Sciences, Novosibirsk, Russia; Siberian Federal Scientific Centre of Agro-BioTechnologies of the Russian Academy of Sciences, Krasnoobsk, Novosibirsk Region, Russia

**Keywords:** spider mites, species identification, allozyme analysis, MALDI-TOF MS, DNA barcoding, PCR-RFLP, ITS, COI, паутинные клещи, молекулярная идентификация, аллозимный анализ, MALDI-TOF MS, ДНК-штрихкодирование, ПЦР-ПДРФ, ITS, COI

## Abstract

Spider mites (Acari: Tetranychidae) are dangerous pests of agricultural and ornamental crops, the most economically significant of them belonging to the genera Tetranychus, Eutetranychus, Oligonychus and Panonychus. The expansion of the distribution areas, the increased harmfulness and dangerous status of certain species in the family Tetranychidae and their invasion of new regions pose a serious threat to the phytosanitary status of agro- and biocenoses. Various approaches to acarofauna species diagnosis determine a rather diverse range of currently existing methods generally described in this review. Identification of spider mites by morphological traits, which is currently considered the main method, is complicated due to the complexity of preparing biomaterials for diagnosis and a limited number of diagnostic signs. In this regard, biochemical and molecular genetic methods such as allozyme analysis, DNA barcoding, restriction fragment length polymorphism (PCR-RFLP), selection of species-specific primers and real-time PCR are becoming important. In the review, close attention is paid to the successful use of these methods for species discrimination in the mites of the subfamily Tetranychinae. For some species, e. g., the two-spotted spider mite (Tetranychus urticae), a range of identification methods has been developed – from allozyme analysis to loop isothermal amplification (LAMP), while for many other species a much smaller variety of approaches is available. The greatest accuracy in the identification of spider mites can be achieved using a combination of several methods, e. g., examination of morphological features and one of the molecular approaches (DNA barcoding, PCR-RFLP, etc.). This review may be useful to specialists who are in search of an effective system for spider mite species identification as well as when developing new test systems relevant to specific plant crops or a specific region

## Abstract (ru)

Паутинные клещи (Acari: Tetranychidae) являются опасными вредителями сельскохозяйственных и декоративных культур; наиболее экономически значимые из них относятся к родам Tetranychus, Eutetranychus, Oligonychus и Panonychus. Расширение ареалов распространения, усиление вредоносности и статуса по степени опасности отдельных видов тетранихид, инвазия опасных вредителей в новые регионы представляют серьезную угрозу для фитосанитарного состояния агро- и биоценозов. Различные подходы к видовой диагностике акарофауны определяют достаточно разнообразный спектр существующих в настоящее время методов, общая информация по которым приведена в данном обзоре. Идентификация паутинных клещей по морфологическим признакам, считающаяся основным методом, осложнена вследствие трудоемкости подготовки биоматериала для диагностики и ограниченного числа диагностических признаков. В связи с этим важное значение приобретают биохимические и молекулярно-генетические методы, такие как аллозимный анализ, ДНК-штрихкодирование, полиморфизм длины рестрикционного фрагмента (ПЦР-ПДРФ), подбор видоспецифичных праймеров, ПЦР в реальном времени и изотермическая амплификация. В обзоре пристальное внимание уделено успехам применения этих методов для видовой дискриминации клещей подсемейства Tetranychinae. Для некоторых видов, например обыкновенного паутинного клеща (Tetranychus urticae), разработан спектр методов идентификации – от аллозимного анализа до петлевой изотермической амплификации (LAMP), тогда как для многих других видов доступно гораздо меньшее разнообразие подходов. Наибольшей точности в определении паутинных клещей можно добиться, используя комбинацию нескольких методов, например осмотр морфологических признаков и один из молекулярных подходов (ДНК-штрихкодирование, ПЦР-ПДРФ и др.). Обзор может быть полезен специалистам, находящимся в поиске эффективной системы для видовой дискриминации паутинных клещей, а также при разработке новых тест-систем, актуальных для конкретных культур растений или определенного региона.

## Introduction

Herbivorous mites cause significant damage to agricultural and ornamental crops. The harmfulness of the mites is manifested in a decrease in yield and deterioration in the quality of crop production; leads to a decrease in drought resistance and winter hardiness as well as to the loss of decorative properties of cultivated plants (Devi et al., 2019; Chaires-Grijalva et al., 2021; Obasa et al., 2022; Ulyanova et al., 2022). Changes in weather and climate conditions in almost all regions of the world, including the Russian Federation, contribute to a wider spread and mass reproduction of pests, transformation of their geographical habitats, changes in the population dynamics and trophic relationships with the host plants (Musolin, Saulich, 2012; Zeynalov, 2017; Koshkin et al., 2021), which fully applies to herbivorous acarofauna (Volkova, Matveikina, 2016; Zeynalov, Orel, 2021), e. g., expansion of habitats, increased harmfulness, and the emergence of populations resistant to acaricides have recently been observed in spider mites (Acari: Tetranychidae), one of the main groups of phytophagous mites (Balykina et al., 2017; Zeynalov, Orel, 2021). The phytosanitary situation is complicated by the high risk of introducing quarantine species with the plant material imported as part of international trade for the purpose of growing, propagating and selling flower and other plant products (Rak, Litvinova, 2010; Petrov et al., 2016; Kamayev, 2018; Kamayev, Mironova, 2018; Vásquez, Colmenárez, 2020).

The Tetranychidae family is subdivided into two subfamilies, Bryobiinae and Tetranychinae, and includes at least 71 genera and more than 1250 described species, 100 of which are dangerous pests (Migeon et al., 2010). The most common species of the Tetranychidae family belong to genera Tetranychus, Eutetranychus, Oligonychus, and Panonychus (Ben-David et al., 2007). Among them, the two-spotted spider mite (Tetranychus urticae Koch.) and European red mite (Panonychus ulmi Koch.) are regarded as the most harmful species (Ben-David et al., 2007). T. urticae is ubiquitous and damages a wide range of crops as well as ornamental woody and herbaceous plants from various botanical families. The habitats of the two-spotted spider mite and other representatives of this family in open-ground agro- and biocenoses have been gradually expanding, covering more and more territories of Russia (Kamayev, Karpun, 2020; Ulyanova et al., 2022). The harmfulness of the spider mites that damage coniferous plants has been increasing as well. Thus in the south of Western Siberia, the spruce spider mite (Oligonychus ununguis Jacobi) damaging the European and Siberian spruces and Siberian fir used for planting greenery in urban areas has become a threat (Ulyanova et al., 2022). The risk of importing quarantine pests with planting material has also increased, e. g., the sugi spider mite (Oligonychus hondoensis Ehara), invasive for this territory, was found in the Krasnodar Region (Kamayev, Karpun, 2020).

Correct identification of spider mite species is of great scientific and practical importance for the study of their population dynamics and timely control of their numbers in agro- and biocenoses as well as for the elimination of international plant quarantine-based trade barriers (Li et al., 2015). Currently, several methods are in use to diagnose the members of the Tetranychidae family, including identification by the morphological characteristics of adult as well as biochemical (protein-based) and molecular (DNAbased) methods. The objective of this review is to consider the modern methods and approaches used to identify the most common species of spider mites (Tetranychidae: Tetranychinae).

## Morphological methods

At present, the identification of herbivorous mites is carried out mainly by the traditional methods based on visual examination of morphological features. The foundations of the morphological method in determining spider mite were laid in the former USSR by prominent scientists V.I. Mitrofanov, I.Z. Livshits and Z.I. Strunkova who hugely contributed to the establishment of a whole area of scientific research devoted to species Tetranychidae family diagnosis (Mitrofanov et al., 1987). The approach was further developed in the research and applied works by S.Ya. Popov et al. (Popov, 2013), A.K. Akhatov (2016) et al., many of which still remain relevant

Determination of the mite’s genus and species is usually based on the shape and size of male genitalia (Morphological Identification…, 2014). However, their microscopic size, slight differences in diagnostic features in the species belonging to the same genus, and the laboriousness of preparing the biomaterial for analysis significantly complicate their morphological identification (Konoplev et al., 2017). Another drawback of this approach is the impossibility of determining the species based on other stages of their development (eggs, larvae, nymphs), since diagnostic differences exist only in adults. Moreover, for some closely related species, morphological identification is almost impossible, e. g., genus Amphitetranychus includes three species and only one of them, A. viennensis, can be recognized by the shape of the aedeagus, while A. quercivorus and A. savenkoae are difficult to separate based on this trait (Arabuli et al., 2019).

The slides are prepared using modified Faure–Berlese media, of which Hoyer’s medium (Walter, Krantz, 2009) is the most common for fixing herbivorous mites and consists of 50 ml of distilled water, 30 ml of gum arabic, 200 ml of chloral hydrate, 20 ml of glycerin. Mites are placed in a drop of medium on a glass slide and covered with a coverslip. The slide is heated at temperatures of 40–60 °С with exposure time varying from 24 hours to 5–10 days, or 3 hours at 70–85 °С (Kamayev, 2019), which contributes to the clarification and straightening of the mite, so its proper diagnosis can be performed. If slides are to be stored for a long time, one is recommended to dry them additionally in a thermostat at a temperature of 40–45 °С for 5–7 days. After drying, the edges of the coverslips are filled with varnish, so the slides can be stored indefinitely. Mite species are diagnosed in transmitted light using a phase-contrast microscope at a 10 to 1000-time magnification.

In general, the morphological method requires considerable time and is very demanding in terms of qualification and experience of those who apply it. In some cases, morphological identification is supplemented by ecological and behavioral responses of the species, as well as by information on the host plant on which the species has been found, which can facilitate species identification (Akhatov, 2016). Some features of the life cycle, such as the diapausing phase, wintering sites, diapause exit time, concentration on certain organs of host plants, and the specificity of damage symptoms, can also play an important role in the species diagnosis

## Species crossing

Genetic incompatibility when crossing closely related species of tetranychid mites is considered one of the best criteria for their discrimination. The reproductive barriers can be caused either by the morphological features (the size of the aedeagus knob exceeding the size of the female epigyne) or by the death of zygotes, which affects the viability of eggs or manifests itself in a different sex ratio in the offspring. For instance, the experiments of Russian researchers on crossing between species Tetranychus atlanticus, T. urticae, and T. sawzdargi showed their complete genetic isolation (Popov, 2013). The reproductive isolation of two morphologically related species of Amphitetranychus spp., manifesting itself in the absence of female offspring, was confirmed in the reciprocal crossing of A. savenkoae and A. quercivorus (Arabuli et al., 2019).

According to a number of scientists (Popov, 2013; Arabuli et al., 2019), a combination of the morphological method with the crossing results for closely related or distant species is a reliable criterion for spider mite species diagnosis. Unfortunately, this method is only applicable for scientific purposes, and cannot be used for practical applications.

## Biochemical methods

One of the biochemical methods for identifying species of spider mites is allozyme analysis that is protein electrophoresis enabling for efficient detection of enzyme polymorphisms (Navajas, Fenton, 2000). To perform the analysis, a single individual is homogenized, its protein extract is isolated and subjected to electrophoresis. In the electric field, proteins are separated according to their size and net electrical charge. After electrophoresis, the gel is histochemically stained to detect specific enzymes. Based on the number and arrangement of the stained fractions, one can make judgments about the alleles encoding a given enzyme in each individual (Kutlunina, Ermoshin, 2017). For correct identification, it is important to choose the enzyme system in which there will be no variability in stripe patterns within a species (polymorphic alleles) and common stripes across species (Gotoh et al., 2007).

The analysis has been widely used to identify insects (Turak, Hales, 1994) and ixodid ticks (Lampo et al., 1997). It has also been applied for spider mite identification, using such enzymes as esterase, phosphoglucosemerase, and malate dehydrogenase (Enohara, Amano, 1996; Goka, Takafuji, 1998; Gotoh et al., 2004, 2007; Arabuli et al., 2019). Such spider mite species as Panonychus citri and P. mori were first identified using allozyme analysis of esterases (Osakabe, 1987). Later it was shown that the same method could be used to identify three more species of the Panonychus mites endemic for Japan (Gotoh, 1992) (see the Table). Using esterase zymograms, Gotoh et al. (2007) were able to distinguish females of four Tetranychus species, and based on the analysis of phosphoglucomutase isoenzymes – all 13 Japanese species of genus Tetranychus (see the Table). The allozyme analysis of esterases was also used for species diagnosis of the representatives of genus Amphitetranychus (Arabuli et al., 2019). The obtained zymograms were species-specific and made it possible to confidentially distinguish all three species (see the Table).

**Table 1.**
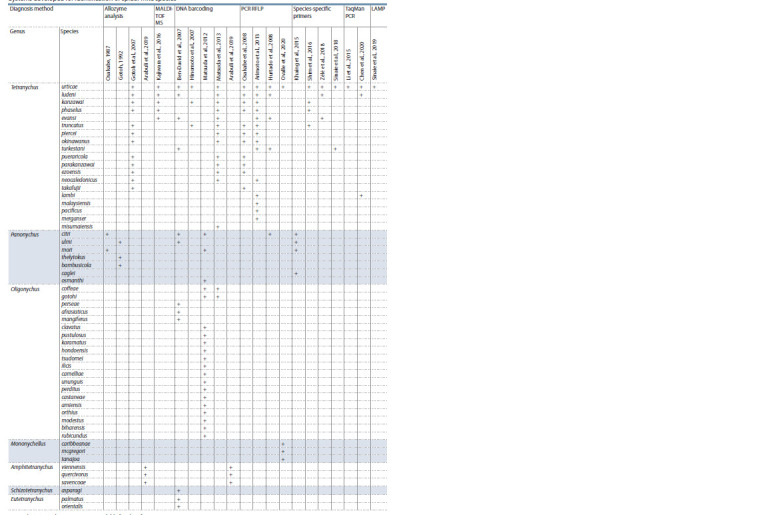
Systems developed for identification of spider mite species Notе. The “+” sign denotes a species available for identification

Thus, allozyme analysis is an effective tool for identifying spider mite species and has a much higher resolution than the morphological method. However, it does not evaluate all possible allele variants present in populations (Altukhov, 2003).

Another biochemical method proposed for the identification of spider mites is matrix-assisted laser desorption/ ionization time-of-flight mass spectrometry (MALDI-TOF MS) (Kajiwara et al., 2016) that has been a well-established method for identifying microbial species (Singhal et al., 2015). In recent years, this method has also been used to investigate plant pests and insects, such as nematodes, Drosophila fruit flies, mosquitoes, etc. (Ahmad et al., 2012).

In this method, species identification is achieved via comparing the spectra of the studied samples with the samples from the spectral database of previously identified organisms. The databases provided by instrument manufacturers currently do not contain reference spectra for plant pests and parasites, but the instrument software usually provides an option to integrate your own reference spectra into existing databases and subsequently perform automated comparisons (Murugaiyan, Roesler, 2017). To create the reference spectra, it is necessary to use the samples whose species have been identified by, e. g., morphological methods

One of the method’s important advantages if compared to other identification techniques is the fast sample preparation and short testing time (Murugaiyan, Roesler, 2017). For diagnosis, it is sufficient having just one individual, whose protein extract must first be isolated using 70 % formic acid and acetonitrile, or the mite can be directly located on the metal target of the device, where formic acid and acetonitrile are added, followed by a matrix solution (Kajiwara et al., 2016). The method’s main disadvantage is the high cost of the device, but the price of one analysis is very low and includes just the cost of the matrix solution and the calibration standard (provided that there is a reusable steel plate for samples).

Kajiwara et al. (2016) showed that closely related species of Tetranychus spider mites (see the Table) had different mass spectra allowing for their identification. The identification was performed by comparing the three main spectral peaks within a m/z range from 2000 to 10 000 corresponding to ribosomal proteins. In adults of both sexes, all three peaks were differentiated, which made it possible to attribute them to the same species. In the case of nymphs, only two of the three main peaks could be identified. Minor peaks were either sex- or developmental stage-specific and were not used for identification (Kajiwara et al., 2016).

In general, MALDI-TOF MS is a promising method for the identification of spider mites, but its application is still limited and requires further research. In particular, there may be spectral differences between geographically different populations, which has been shown for some insects (see Murugaiyan, Roesler, 2017 and references to this article), and it is also assumed that the spectra of one species can be influenced by its host plant (Kajiwara et al., 2016).

## Molecular methods

For the purposes of mite species diagnosis, the molecular methods based on DNA sequencing are used along with biochemical ones. In these methods, the DNA fragments amplified by PCR serve as biological barcodes to identify the species a sample belongs to (Hajibabaei et al., 2007). Both nuclear and mitochondrial genome sequences can be used as such barcodes. Since more and less conserved regions alternate within a ribosomal DNA cluster (genes 18S, 5.8S, 28S rRNA and internal transcribed spacers 1, 2 (ITS1, ITS2), respectively), it becomes possible to select different marker sequences to separate taxa of different ranks. The 18S and 28S rDNA gene sequences can be used to compare phylogenetically distant taxa (Matsuda et al., 2014), and the ITS regions are effective for distinguishing between species and even populations (Hillis, Dixon, 1991; Hurtado et al., 2008). Among the mitochondrial genes, the cytochrome c oxidase subunit I (COI ) gene sequence is used to identify species and analyze phylogenetic relationships. In addition to the Folmer fragment (Barcode of Life (BOLD), Folmer et al., 1994) being the 5′-terminal sequence of the COI gene, other gene sequences are used for barcoding spider mites (Hinomoto et al., 2007; Ros, Breeuwer, 2007; Matsuda et al., 2013; İnak et al., 2022).

In (Ben-David et al., 2007), the authors used the ITS2 sequence as a barcode to distinguish 16 species of spider mites of the Tetranychidae family endemic for Israel (see the Table), and the barcode sequence of each type was uniquely distinguishable from all others. The effectiveness of the DNA barcoding for species identification was also confirmed by COI gene fragment-based identification of the spider mite species collected from agricultural fields in Vietnam (Hinomoto et al., 2007) (see the Table).

The results also demonstrated the method’s limitations, e. g., some samples could not be classified due to lack of sequence information in the databases. Other species, such as T. urticae and T. turkestani, T. neocaledonicus and T. glovery, could not be uniquely identified by their DNA sequences. The impossibility to distinguish between T. urticae and T. turkestani based on the COI gene sequence was also confirmed in the mites collected in different regions of Russia and Turkey (Konoplev et al., 2017; İnak et al., 2022), which can be overcome by further accumulation of data on the sequences of different DNA regions from morphologically identified samples. Matsuda et al. (2013) identified the mite species of genus Tetranychus by sequencing the ITS and COI genes. The authors concluded that 10 out of 13 species of the Japanese mites of genus Tetranychus can be identified using the ITS sequence, while the COI gene sequence made it possible to identify all 13 species (see the Table).

In contrast to genus Tetranychus, where some species cannot be distinguished based on DNA sequences, this method proved to be effective for genus Oligonychus (Matsuda et al., 2012), so all 17 Japanese species were successfully distinguished based on any of the sequences (COI, ITS, and 28S rDNA), including those that were difficult to differentiate by morphological characters, such as O. castaneae and O. coffeae (see the Table). Molecular analysis of the COI gene sequence of the mites of genus Amphitetranychus showed that the sequence is effective for the identification of all three species (Arabuli et al., 2019) (see the Table).

Thus, the DNA barcoding is an effective tool for the identification of individual genera of spider mites (such as Oligonychus and Amphitetranychus). However, some closely related species (e. g., from genus Tetranychus) may be indistinguishable from each other by their barcode sequences, as it has been shown for T. kanzawai, T. parakanzawai, and T. ezoensis having similar ITS sequences (Matsuda et al., 2013); and T. urticae and T. turkestani having similar COI gene sequences (Hinomoto et al., 2007; Konoplev et al., 2017). For these species, other marker genes should be selected.

The desire to reduce the time spent for analysis has led to the development of faster methods for identifying spider mites, one of these being PCR-restriction fragment length polymorphism (PCR-RFLP). In this method, fragments of genomic DNA (nuclear or mitochondrial), amplified by PCR, are subjected to hydrolysis by restriction endonucleases. The restriction products are subjected to gel electrophoresis, so a conclusion can be made about the presence or absence of a restriction site in this sample and, as a result, a species can be identified.

PCR-RFLP is widely used for species identification of various organisms (Ratcliffe et al., 2003; Han et al., 2004; Alam et al., 2007). In spider mites, the method was first applied to distinguish between three species of genus Panonychus and T. urticae (Osakabe, Sakagami, 1994) (see the Table). Later, Gotoh et al. (1998) were able to differentiate T. urticae and T. pueraricola by applying PCR-RFLP to the ITS2 region. In (Hurtado et al., 2008), the authors applied the method to the ITS fragment (ITS1, 5.8S rDNA, ITS2) to identify five species of spider mites that inhabited Spanish citrus orchards (see the Table). The most harmful was the common spider mite (T. urticae) that made it necessary to quickly distinguish it among the others, which was achieved through the RsaI enzyme patterning the restriction fragments, while hydrolysis with two other enzymes made it possible to identify the other four species (Hurtado et al., 2008). The PCR-RFLP applied to ITS regions is used in the Japanese Imported Plant Quarantine Department (Arimoto et al., 2013; Li et al., 2015) and enables one to detect all 14 spider mite species found in Japan, including five exotic ones (Osakabe et al., 2002, 2008; Arimoto et al., 2013) (see the Table). Meanwhile, the PCR-RFLP applied to the COI region was successfully used to identify four spider mite species thriving on cassava in Colombia (Ovalle et al., 2020) (see the Table).

PCR-RFLP is a relatively inexpensive and effective method for spider mite identification, used in many countries around the world (Hurtado et al., 2008; Arimoto et al., 2013; Ovalle et al., 2020). However, it can only be applied to those species it has been developed for, e. g., to identify mites in an area whose species composition is well established. When new species are added to the system, their RFLP pattern should be analyzed and, if the pattern matches the existing ones, the system should be modified by selecting other diagnostic restrictases. The RFLP pattern can also vary in different populations of the same species due to nucleotide substitutions at the restriction site (Arimoto et al., 2013).

Another approach for rapid mite identification has been selection of species-specific primers. The DNA regions where such primers are defined should be common for the species being determined, but the sequences themselves should differ between species of the same genus (Shim et al., 2016). In this case, both whole pairs of species-specific primers can be used, as well as a combination of one universal primer and the second primer being unique for a particular species. Such primers increase the accuracy and speed of PCR-based identification of spider mite species, but their development is not a trivial task. Khaing et al. (2014) succeeded in selecting pairs of species-specific primers for the ITS2 region for four species of spider mites belonging to genus Panonychus (see the Table). Later, species- specific primers were developed for the spider mites of genus Tetranychus (see the Table) common in Korea and very similar morphologically (Shim et al., 2016).

Combining several species-specific primers in a single tube allows applying the multiplex approach to identify several species at once. In this case, the melting temperature of the primers should be high, and the resulting amplicons – short, but different in length. Additionally, multiplex PCR requires selecting amplification conditions, such as the ratio of primer pairs and the elongation time (Zélé et al., 2018). Zélé et al. (2018) designed and successfully multiplexed primers complementary to the ribosomal locus in a single reaction to identify the most common spider mites found in southwestern Europe (see the Table). Multiplex PCR has also been used to discriminate between the two main spider mite species, T. urticae and T. turkestani found in Iranian greenhouses (Sinaie et al., 2018).

Using real-time PCR for species identification significantly reduces the time for analysis because this method does not require gel electrophoresis since the accumulation of the PCR product is monitored directly during the reaction using optical sensors built into the cycler. Two types of labels are used to detect a PCR product: intercalating agents (e. g., SYBR Green) or modified oligonucleotides containing fluorophores (DNA probes) (Bikbulatova et al., 2012). In addition, real-time PCR analysis combines primer annealing and elongation steps, resulting in a shorter reaction time than conventional PCR (Li et al., 2015).

Li et al. (2015) developed the TaqMan PCR detection system to distinguish T. urticae among other closely related species that has proved to be highly specific and reliable. TaqMan is one of hybridization DNA probes – an oligonucleotide complementary to the amplified internal region of the DNA fragment, labeled at the ends with fluorophores – being a reporter and a quencher, respectively. When they are on the same probe, the quencher absorbs the signal from the reporter. During amplification, the polymerase moving along the DNA destroys the probe, so the reporter and quencher move away from each other, and the reporter’s fluorescence becomes noticeable (Bikbulatova et al., 2012). The species-specific primers and probe were designed for T. urticae’s ITS1 sequence, since it contained more intraspecific polymorphisms than the ITS2 sequence widely used for Tetranychus species phylogeny and identification (Li et al., 2015).

Another team (Chen et al., 2020) developed TaqMan species-specific probes for the identification of spider mites thriving in the cotton fields of Australia (see the Table), and selected the conditions for their use in one test tube (multiplex approach). An important feature of this approach was the possibility of extending it to other types of spider mites with minor modification. Since it used a pair of primers universal for spider mites for amplification, it was necessary, when adding another species to the test system, to develop and add a species-specific probe for it and select reaction conditions (Chen et al., 2020). The authors used DNA probes for three species of mites in one reaction and considered it was possible to increase their number to five (add or replace with DNA probes for the required species) to diagnose up to five different mite species with one PCR.

Another method that can be used for species identification purposes is loop mediated isothermal amplification (LAMP) (Tomita et al., 2008). Unlike the classical PCR, LAMP uses a different thermostable polymerase with high displacement capacity that can itself displace the second strand without thermal denaturation. That is why LAMP reaction takes place at the same temperature (60–65 °C) and does not require an DNA amplifier. Another feature of LAMP is that not two, but four or six primers are used during the reaction, which determines its high specificity (Notomi et al., 2000). On the other hand, selecting such primers for LAMP is a rather laborious task. The reaction’s product can be detected in several ways, including the naked eye after adding a fluorescent dye to confirm the reaction has taken place (Tomita et al., 2008). Thus, this method not only does not require special equipment or specially trained specialists, but is also suitable for largescale field studies (Hsieh et al., 2012; Sinaie et al., 2019).

As a well-established technique, LAMP has been used in a wide range of applications, including the identification of plant pathogens and insect species (Ahuja, Somvanshi, 2021; Dermauw et al., 2022). For the identification of the common spider mite, a highly sensitive method combining PCR and LAMP (PCR-LAMP) has been developed (Sinaie et al., 2019). It includes the standard PCR performed prior to isothermal amplification to reduce the false negatives and increase the sensitivity of the LAMP assay. The authors have shown that PCR-LAMP is a fast and reliable method for T. urticae biomaterial detection

## Conclusion

Our review of currently available methods for diagnosing the most significant species of herbivorous mites (Tetranychidae: Tetranychinae) has demonstrated that a wide variety of approaches have been accumulated that can be used by choice, depending on the goals of species identification. The morphological methods, despite the complexity of required preliminary operations to prepare mites for analysis, still remain the main ones in determining this group of phytophages. However, the new approaches developed in recent decades and based on biochemical and molecular markers open up even greater opportunities for faster and more accurate identification of species. For instance, some species of spider mites such as T. urticae can be properly diagnosed using one of the molecular approaches (DNA barcoding, PCR-RFLP, TaqMan PCR, etc.). At the same time, some other species have not yet been diagnosed based on their DNA sequences. In such cases, the use of several identification methods at once is justified. Such an integrated approach was applied to the diagnosis of the species of genus Amphitetranychus (Arabuli et al., 2019) whose morphological characteristics were supplemented by biochemical, and molecular ones and verified in crossing experiments. It is important to note that several approaches can be implemented on the same individual, provided that DNA extraction methods are used without destroying the sample (Khaing et al., 2013; Shim et al., 2016) or a whole mite is used as a PCR matrix (direct PCR) (Sakamoto, Gotoh, 2017), and for subsequent morphological characterization. It has been shown that, after such manipulations, specimens retain their morphological features intact, including dorsal setae and aedeagus (Sakamoto, Gotoh, 2017).

## Conflict of interest

The authors declare no conflict of interest.
